# Air sampling accurately captures circulating zoonotic viral diversity emerging from poultry live-animal markets

**DOI:** 10.21203/rs.3.rs-5682962/v1

**Published:** 2025-02-13

**Authors:** Peter Cronin, Jurre Siegers, Vireak Heang, Songha Tok, Sarath Sin, Benjamin Sievers, Victor Omondi, Sithun Nuon, Kimtuo Chhel, Janin Nouhin, Vutha Chim, Bunnary Seng, Makara Hak, Sorn San, Sothyra Tum, Filip Claes, Cadhla Firth, Yvonne Su, Gavin Smith, Erik Karlsson

**Affiliations:** Programme in Emerging Infectious Diseases, Duke-National University of Singapore Medical School, Singapore; Institut Pasteur du Cambodge; Virology Unit, Institute Pasteur du Cambodge, Pasteur Network, Phnom Penh, Cambodia; Virology Unit, Institute Pasteur du Cambodge, Pasteur Network, Phnom Penh, Cambodia; Virology Unit, Institute Pasteur du Cambodge, Pasteur Network, Phnom Penh, Cambodia; J. Craig Venter Institute; Virology Unit, Institute Pasteur du Cambodge, Pasteur Network, Phnom Penh, Cambodia; Virology Unit, Institute Pasteur du Cambodge, Pasteur Network, Phnom Penh, Cambodia; Virology Unit, Institute Pasteur du Cambodge, Pasteur Network, Phnom Penh, Cambodia; Institut Pasteur in Cambodia; National Animal Health and Production Institute, Phnom Penh, Cambodia; National Animal Health and Production Institute, Phnom Penh, Cambodia; Food and Agriculture Organization of the United Nations, Emergency Center for Transboundary Animal Diseases, Country Office, Phnom Penh, Cambodia; National Animal Health and Production Institute, Phnom Penh, Cambodia; National Animal Health and Production Institute, Phnom Penh, Cambodia; Food and Agriculture Organization of the United Nations, Emergency Center for Transboundary Animal Diseases, Regional Office for Asia Pacific, Bangkok, Thailand; College of Public Health, Medical and Veterinary Sciences, James Cook University, Townsville, Queensland, Australia; Duke-NUS Medical School; Duke-NUS Medical School; Institut Pasteur du Cambodge

## Abstract

Environmental surveillance has emerged as a pivotal strategy for early detection of pathogens that pose threats to humans (1) but has not been utilized for zoonotic agents. In Asia, live-bird markets (LBMs) are key human-animal interfaces for zoonotic virus transmission (2). Traditional sampling strategies are time-consuming, expensive, threaten animal welfare and have significant occupational biosafety risks. In this study, we assessed the performance of metagenomics on environmental samples (ES) compared to traditional poultry swabs for detecting avian viral pathogens in LBMs in Cambodia. ES, including air, cage swabs, and carcass wash water, were collected alongside throat and cloacal swabs from domestic chickens and ducks across twelve sampling visits in two LBMs over a 15-month period. Viral nucleic acids were extracted and sequenced using a capture probe-based metagenomics approach. Our results show that metagenomics on ES outperformed traditional poultry samples in detecting the highly pathogenic Influenza A/H5N1, including circulating clades 2.3.4.4b and 2.3.2.1c, which were found in the environment but missed by poultry swabs on multiple occasions. Environmental metagenomics was also highly sensitive in the detection of over 40 other viruses from key pathogen families such as *Astroviridae*, *Coronaviridae*, *Picornaviridae*, and *Retroviridae*. Viral contigs from ES showed high similarity to those from poultry swabs further highlighting the accuracy of this approach. Our findings highlight metagenomics on ES can precisely and effectively replicate metagenomic results from traditional surveillance samples, offering broader coverage and enhanced detection of avian pathogens. This robust approach could be pivotal for mitigating zoonotic spillover, controlling pathogen transmission at LBMs, and enhancing pandemic preparedness strategies.

## Introduction

Environmental surveillance has become an increasingly important strategy for early detection and monitoring of viral pathogens (1, 3). Several major disease outbreaks in humans have resulted from zoonotic spillover from animals (4–9) making pathogen surveillance at human-animal interfaces such as live-bird markets (LBMs) critical. LBMs are common in Asia, holding critical socio-economic significance. However, they are also marked by the prevalence of both endemic and emerging infectious diseases of One Health importance, including highly pathogenic avian influenza A virus (AIV) H5N1 (2, 10, 11). Highly pathogenic A/H5N1 has a mortality rate exceeding 50% in humans (12, 13) while also imposing a significant economic burden on global poultry industries (14, 15). Outbreaks of pathogens like A/H5N1 in poultry often lead to severe economic losses affecting livelihoods reliant on small scale poultry production (16–18). Other pathogenic avian viruses, including members of the *Rotavirus* (19, 20), *Avastrovirus* (21), *Gammacoronavirus* (22) and *Alpharetrovirus* genera (23) also cause substantial losses in domestic poultry. Improving pathogen surveillance systems at LBMs is essential to infectious disease monitoring, risk mitigation, and outbreak response, especially in developing regions of the world.

Pathogen monitoring at LBMs has traditionally relied on random testing of individual animals via oropharyngeal and cloacal swabs. However, this approach may miss circulating pathogens if the selected bird is not infected when sampled, thus limiting the probability of detection. In addition, traditional sampling strategies are time-consuming, costly, and pose significant occupational biosafety risks while compromising animal welfare (24, 25). ES, particularly air, can provide a more comprehensive, safe, and easier approach to pathogen surveillance. Furthermore, it is hypothesized that poultry virus shedding can result in contamination of the surrounding environment, with the environment becoming a reservoir of pathogens exacerbated by insufficient biosecurity measures at LBMs (26, 27). Previously, we demonstrated through air sampling that workers at an LBM in Cambodia were exposed to influenza subtypes A/H5N1 and A/H9N2 (28) and this approach has also proven successful in accurately detecting pathogens in other settings (29–32). In addition, ES can be used to detect influenza A virus subtypes from sources such as the wash water used to clean bird carcasses after de-feathering at LBMs (28).

We hypothesize that ES could supplement traditional poultry pathogen surveillance and provide insight into risks of environmental exposure for LBM workers and consumers. This information is crucial for mitigating risks of pathogen transmission between birds and throughout the poultry supply chain, and for preventing spillover to humans. However, it remains unknown whether metagenomic data from ES in LBMs accurately reflects data from traditional poultry samples used for pathogen surveillance.

To date, environmental pathogen surveillance has largely relied on PCR-based methods, often used for monitoring of select human pathogens (e.g., influenza A and SARS-CoV-2) in wastewater and sewage (33, 34). Although these approaches offer high sensitivity and specificity, they are limited in the range of viruses they can detect. Unbiased metagenomics has emerged as a method with a much broader target range for virus identification and surveillance, allowing detection of a diverse array of known and novel pathogens (35). One major challenge using metagenomics is the low amount of viral nucleic acid present in most sample types, compared to higher levels of host and environmental nucleic material. Methods to reduce non-target sequences in metagenomic data have been explored, but vary in effectiveness (36). However, viral enrichment using probe hybridization methods has been shown to significantly improve sensitivity across different sample types, enabling the accurate detection of low abundance and/or low-frequency virus variants (37–40).

In this study, we compared the performance of targeted virus capture probe-based metagenomics on several different ES types versus traditional poultry swab samples from LBMs for the detection and surveillance of avian pathogens. We hypothesize that using metagenomics on ES may offer a more robust approach to facilitate the early detection of virus transmission at high-risk human-animal interfaces, allowing for more rapid and effective risk mitigation measures to take place.

## Materials and methods

### Study design and sample collection

Between January 2022 and April 2023 samples were collected on 12 occasions in Cambodia at Orussey market, Phnom Penh province and Daun Keo market, Takeo province (Supplementary Fig. 1). Individual animal samples consisted of oropharyngeal and cloacal swabs collected from an domestic ducks and chickens. These were combined into pools of ten individual cloacal or oropharyngeal swabs for sample processing, with ducks and chickens pooled separately. ES comprised air, carcass wash water, cage swabs, and drinking water. Air samples were collected for three hours at three locations at each market (the slaughter area, the poultry holding/storage area, and a “control area” - either the food storage area located in a separate building or > 50m away from the LBM) using AerosolSenseTM Samplers (Thermo Fisher Scientific). Air was sampled through an omnidirectional inlet at a rate of 200 L/min. Collected material was eluted from each air sampler by rinsing and vortexing the collection matrix in 2 mL of viral transport medium. Carcass wash water (50 mL) used to wash poultry carcasses after slaughter and de-feathering processes was collected from the slaughter area. Cage swabs were collected from selected cages in the poultry holding area of the market. Where available, 2 mL of poultry drinking water from inside poultry cages was collected. All samples were transported at 4°C to Institut Pasteur du Cambodge, aliquoted into tubes on arrival and stored at − 80°C until further processing.

### RNA extraction, library preparation and next-generation sequencing

RNA was extracted from each sample pool using the Direct-zol RNA Miniprep Kit (Zymo Research, California, USA) according to the manufacturer’s protocol and eluted in 50uL of nuclease-free water. Sequencing libraries were generated using the Twist Library Preparation Enzymatic Fragmentation (EF) kit 2.0 (Twist Bioscience, San Francisco, California, USA) and the Twist Comprehensive Viral Research Panel (CVRP), according to the manufacturer’s protocol (Twist Total Nucleic Acids Library Preparation EF Kit 2.0 for Viral Pathogen Detection and Characterization Protocol and Twist Target Enrichment Standard Hybridization v1 protocol). Briefly, extracted RNA was converted to cDNA using the ProtoScript II First Strand cDNA Synthesis kit (New England Biolabs, Ipswich, Massachusetts, USA) and Random Primer 6 (S1230S) from New England Biolabs (NEB). The NEB Next Ultra II Non-Directional RNA second Strand Synthesis kit (E6111S) was subsequently used to convert the single-stranded cDNA to double stranded DNA (dsDNA). Illumina TruSeq-compatible libraries were generated using the Twist Library Preparation EF kit 2.0 and combined into pools of six libraries. The pooled libraries were enriched by hybridization capture using the Twist Comprehensive Viral Research Panel (CVRP). Following enrichment, libraries were pooled in equimolar ratios, diluted and denatured prior to sequencing according to the standard MiSeq System Denature and Dilute Libraries Guide (Document # 15039740v10). Sequencing was performed Finally, the library was sequenced to generate paired-end 75bp reads using a MiSeq V3 reagent kit (Illumina, MS-102–3001) on the Illumina MiSeq System.

### Processing of viral sequencing data

Raw sequencing data was processed using the CZ ID pipeline (https://czid.org) which is an open-source cloud-based metagenomics pipeline for global pathogen detection and monitoring (41). Viral abundance was estimated as reads per million (RPM) relative to the total number of reads which mapped to virus sequences, while *de novo* assembled contigs were directly downloaded from the CZ ID website. SeqKit was used for sequence length filtering and FASTA file manipulation (42). Contigs *de novo* assembled from poultry swabs were mapped to those assembled in environmental samples using BWA-MEM2 (43). Alignment rates (percentage reads mapped) were calculated using Samtools (44).

### Biostatistical analysis

All biostatistical analyses were carried out in Rstudio (version 4.4.0) (45). Β-diversity (Jaccard Index) was calculated using the vegdist function in the vegan package (version 2.7) (46). To statistically test for differences between sample types we employed a Permutational Analysis of Variance (PERMANOVA) test which was calculated using the adonis function also in the vegan package. Pairwise PERMANOVAs were calculated using the pairwise.adonis function. To visualise virome composition between sample types we used principal coordinate analysis (PCoA) using the dudi.pco function of the ade4 package (version 1.7) (47). To perform a quantitative comparative evaluation of virome composition we employed a median centroid testing methodology based on the Jaccard distances from a PCoA. This approach sees the median PCo1 and PCo2 coordinates calculated (median centroid) for each poultry swab sample type and the distance of all other samples from this point is subsequently determined. Spearman correlations were calculated using the corr.test function in the psych package (48). In order to estimate the proportion of viruses in ES that are likely to have come from each poultry swab type source tracking analysis was conducted using the R package SourceTracker (49). All plots used to visualise data were produced using the ggplot2 package in R (50). Heatmaps were produced using either the ComplexHeatmap package(51) or the circlize package (52). Statistical significance was determined using the non-parametric Wilcox test between two comparative groups. When statistical significance was being determined between three or more comparative groups the non-parametric Kruskall-Wallis with Dunns post-hoc test was employed using the PMCMRplus package. All P-values obtained were corrected for false-discovery rate (FDR) using the Benjamini-Hochberg method (p.adjust function in R). P values are annotated as follows unless otherwise stated: P < 0.05 *; P < 0.01 **; P < 0.001***.

### Phylogenetic tree reconstruction

For phylogenetic tree reconstruction of influenza gene segments from *de novo* assembled contigs all available reference sequences were downloaded from the NCBI Influenza database and GISAID from January 1, 2014 to July 1, 2024. From our metagenomic data, only influenza contigs *de novo* assembled through the CZ ID pipeline and greater than 200bp were included for analysis. Contig taxonomy was determined using blast (53). Reference datasets were subsampled to 500 sequences using SMOT(54) and combined with sequences identified in the current study. For all other viruses, all available full length reference sequences of a given virus were downloaded from the NCBI virus database. Multiple nucleotide sequence alignments were carried out using Clustal Omega using default settings (55). The resulting multiple sequence alignments were manually curated, and erroneous or ambiguous regions were trimmed to ensure alignment accuracy using trimAl (53, 56). Maximum likelihood phylogenies for each gene segment or genome were individually reconstructed using IQ-TREE (version 2.1.4)(57) with the best fit nucleotide substitution model. No bootstrapping or other estimates of nodal support were applied. Phylogenetic trees were plotted in R using the ggtree package (58).

## Results

### Environmental metagenomics accurately detects circulating pathogens of domestic poultry

**Figure 1:**
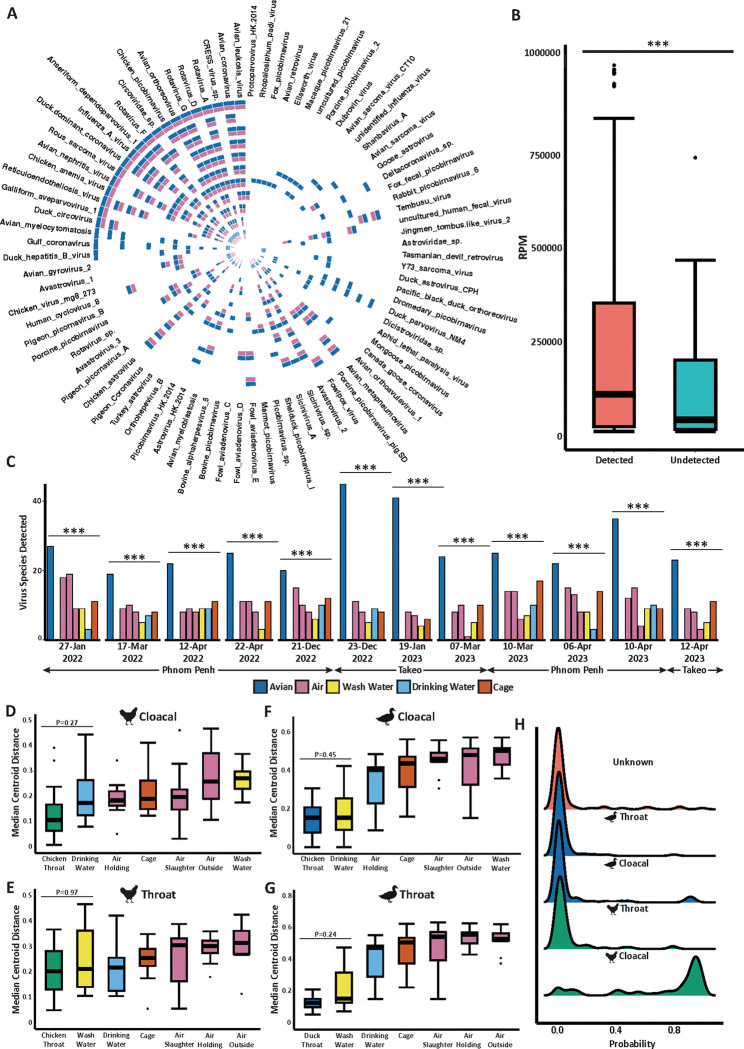
Environmental metagenomics accurately detects circulating poultry pathogens. A) Circular heatmap depicts virus detection between poultry swabs and the environment. Whereby a given virus was found in at least poultry sample a blue cell is shown and whereby that same virus was found in at least one environmental sample at the same time and location an adjacent pink cell is shown. Each track of the heatmap represents one visit to an LBM. B) Boxplot showing the difference in virus abundance between taxa that were successfully detected or undetected from panel A. Statistics were calculated using a Wilcox test. C) Bar plot showing the total number of unique viruses detected in poultry swabs compared to each individual environmental sample at each sampling date. Statistics were calculated using Fishers exact test best all groups. Based on PCoA in Supplementary Figure 5, we calculated the distance of all samples from the median centroid coordinates of D) cloacal and E) oropharyngeal swabs obtained from domestic chickens as well as F) cloacal and G) throat swabs obtained from domestic ducks. Statistics were calculated using a Kruskall-Wallis with Dunns post-hoc test. H) Ridge plot showing the probability of each poultry swab being the source of pathogenic viruses for all environmental samples. Statistics were calculated using a Kruskall-Wallis with Dunns post-hoc test. All P-values obtained were corrected for false-discovery rate (FDR) using the Benjamini-Hochberg method. P values are annotated as follows: P < 0.05 *; P < 0.01 **; P < 0.001***.

To evaluate the performance of metagenomics for viral pathogen detection using ES compared to poultry swabs, we first calculated the detection rates of each viral pathogen among both sample types. A total of 84 viruses were found at least once in domestic poultry swabs across the 12 sampling dates at the two LBMs in Cambodia (Fig. 1A). We showed that the majority of viruses found in poultry swabs could also be found in corresponding ES at each timepoint with detection rates consistently ranging between 70% and 90% (Fig. 1A and Supplementary Fig. 2A and 2B). Common avian respiratory viruses including avian influenza and avian coronavirus were frequently identified in ES (Fig. 1A). While this was also true for other pathogens including Rous sarcoma virus, avian leukosis virus, avian orthoreovirus (*Retroviridae*), chicken astrovirus (*Astroviridae*). Virus sequences that were not be detected in ES were in significantly lower abundance in poultry swabs (Fig. 1B) and were in circulation at the LBMs less often than those successfully detected (Supplementary Fig. 3). Only two respiratory viruses were not found in the environment that were found in poultry swabs (Gull coronavirus, and *Deltacoronavirus* sp.). Other undetected viruses either infect the gastrointestinal tract (viruses in the *Astroviridae* families) or were oncoviruses (including avian sarcoma virus CT10, Y73 sarcoma virus and avian retrovirus) (Fig. 1A). Importantly, there was no association between taxonomic family and detection in ES. For example, although avastrovirus 3 and astrovirus HK-2014 could not be found in the environment, several other viruses in the *Astroviridae* family were successfully identified in both poultry swabs and ES. In addition, we observed remarkable consistency in the performance of environmental metagenomic surveillance at the LBM. Taxa detected in poultry swabs and the environment were consistently co-detected regardless of when and where they were circulating.

We next evaluated the performance of each individual environmental sample type in the detection of corresponding poultry viruses. Air samples collected from the slaughter and holding area as well as cage swabs contained significantly more avian viruses than air from outside the market, wash water or drinking water (Supplementary Fig. 4A and 4B). These findings were consistent between both sampling locations. Furthermore, the total number of viruses detected in each individual ES was significantly less than the total number found in poultry swabs at each sampling date (Fig. 1C), suggesting that a variety of sample types are required to accurately capture the most viral viruses from poultry.

Principal coordinate analysis (PCoA) of b-diversity (Jaccard distance) focusing only on the avian viruses identified in this study (Fig. 1A) revealed statistically significant virome separation between the groups after controlling for location and time (PERMANOVA < 0.001*** and R2 = 0.037) (Supplementary Fig. 5). Pairwise PERMANOVA of each individual comparison revealed no significant differences between ES types including cage swabs, drinking water and air samples from the holding area and slaughter area (Supplementary File 1). Interestingly, air samples from outside the LBM, as well as wash water, were significantly different from all other sample types. Both cloacal and oropharyngeal swabs collected from chickens had significantly different virome compositions compared to the same swab types from ducks (Supplementary Fig. 5). Given the observed differences in virome composition between poultry swab types we wanted to evaluate the relationship/performance with each samples type individually. Thus, based on the PCoA in Supplementary Fig. 5, we calculated the median coordinates (PCo1 and PCo2) of each poultry swab (cloacal and throat from both domestic ducks and chickens) and subsequently measured the distance of all samples from each point (Fig. 1D–G). Using this quantitative comparative evaluation of virome composition we show that no significant difference could be detected between cloacal samples from chickens and samples from drinking water, cage swabs as well as air from both the slaughter and holding area (Fig. 1D). Similar findings were observed when comparing throat swabs from chickens with drinking water, cage swabs and wash water, although air samples were a significantly larger distance by this measure (Fig. 1E). Interestingly, no difference could be detected between wash water and throat or cloacal swabs from ducks when comparing their respective distances from each median centroid (Fig. 1F–G).

To better understand why specific viruses were not detected in ES, we first identified the poultry swab type most associated with undetected taxa. To do this, we calculated Spearman correlations between viral abundance (RPM) and the distance from each poultry swab median centroid as calculated in Fig. 1D–G, comparing the results between detected and undetected taxa identified in Fig. 1A. We found that viruses that were not identified through environmental sampling (undetected) were associated with the virome of throat and cloacal duck samples (Supplementary Fig. 6A and 6B). In both cases, undetected viruses tended to have more negative correlations than those successfully detected suggesting their abundance is higher the shorter the distance to the median centroids of duck samples. The opposite trend was reported for cloacal swabs in chickens which had a strong association with taxa successfully detected in poultry swabs (Supplementary Fig. 6C). However, no significant difference could be found for throat swabs in chickens (Supplementary Fig. 6D). Using a Bayesian approach, we conducted source tracking analysis to estimate the proportion of viruses in ES that are likely to have come from each poultry swab type (Fig. 1H). We found that cloacal swabs from chickens were identified as the dominant source of virus composition for all ES. The probability of cloacal swabs from chickens contributing to the environmental virome composition was significantly higher compared to throat swabs from chickens as well as both sample types from ducks (Fig. 1H).

Importantly, ES analysed using targeted metagenomics revealed an additional 50 viruses which were not found through traditional poultry swab sampling (Supplementary Fig. 7). While the majority of these viruses may be from alternative sources (such as arboviruses or porcine viruses), several avian viruses were also detected, including goose aviadenovirus A. duck adenovirus 4, pigeon circovirus, shelduck picobirnavirus II and avian dependoparvovirus 1. Metagenomics on ES also detected the arbovirus dengue virus 3 as well as the common human respiratory pathogen *Rhinovirus A1* (Supplementary Fig. 7). Importantly, this highlights a major advantage of ES in facilitating the detection of viruses which may be missed by traditional sampling methods or circulating pathogens from other sources.

Overall, these findings suggest that the environmental sampling conducted at LBMs performs better at detecting chicken viruses than those infecting ducks, while cloacal-derived chicken viruses appear to be a major source for the environmental pathogens in LBMs.

### Most viral contigs from poultry align to environmental contigs

**Figure 2:**
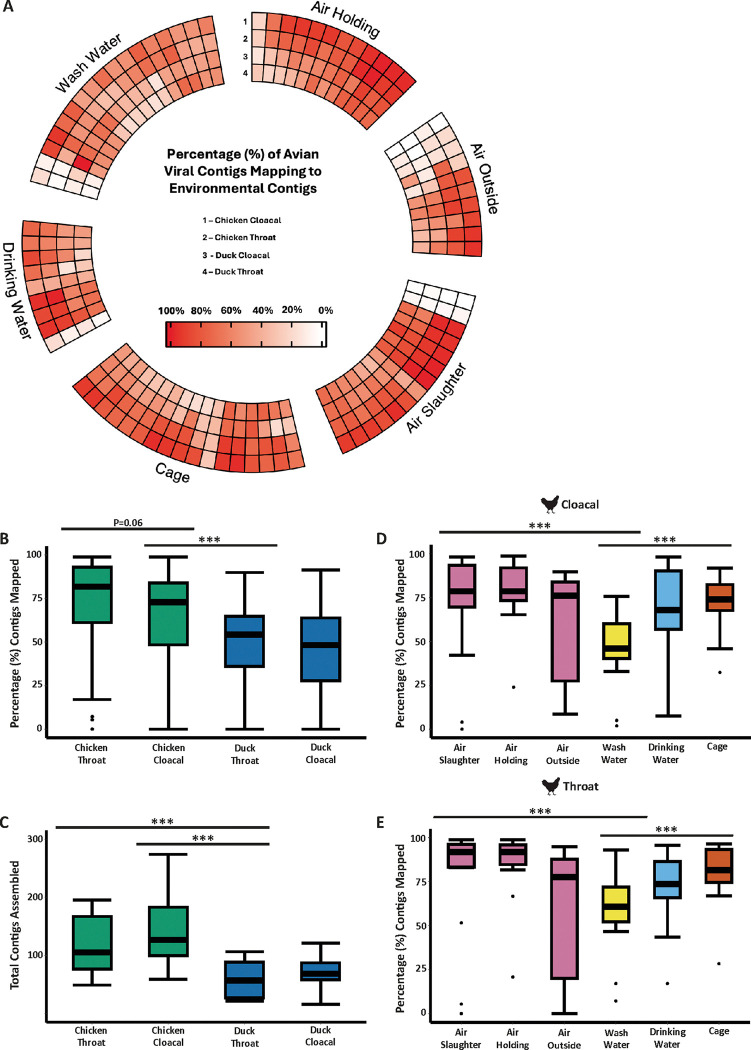
Most avian viral contigs align to environmental contigs. A) Circular heatmap showing the percentage (%) of all avian viral contigs mapping to those assembled from the environment. B) Summary of panel A showing the percentage alignment rate of avian contigs to those *de novo* assembled from environmental samples. C) The total number of contigs assembled for each type of poultry swab collected. Alignment rate for D) cloacal and E) throat swabs collected from chickens is shown for each individual environmental sample. Statistics were calculated using a Kruskall-Wallis with Dunns post-hoc test. All P-values obtained were corrected for false-discovery rate (FDR) using the Benjamini-Hochberg method. P values are annotated as follows: P < 0.05 *; P < 0.01 **; P < 0.001***.

Next, we aimed to assess whether *de novo* assembled viral contigs from ES were an accurate representation of those found in poultry swab samples. We found that the majority of contigs (≥ 200bp) from direct poultry samples mapped to those from ES (Fig. 2A). Interestingly, the proportion of viral contigs between throat and cloacal swabs obtained from chickens were similar (not statistically significant, P = 0.06) (Fig. 2B). However, both sample types had a significantly greater percentage of successfully mapped viral contigs than throat/cloacal swabs obtained from ducks. In addition to having a lower percentage of mapped viral contigs, cloacal and throat samples obtained from ducks yielded significantly fewer viral contigs than those from chickens (Fig. 2C). For both throat and cloacal swabs obtained from chickens, the highest percentage of viral contigs was shown to map to the ES air (slaughter and holding) and cage swabs (Fig. 2D–E). Large variation in the percentage of viral contigs mapped was observed between air samples collected from outside the LBMs (Fig. 2D–E). No significant differences could be determined between the percentage of viral contigs mapped between ES and duck (Supplementary Fig. 8A and 8B). Overall, these findings confirm differences in the performance of ES for different bird species.

### Environmental samples yielded highly pathogenic influenza A contigs more often than poultry swabs

**Figure 3:**
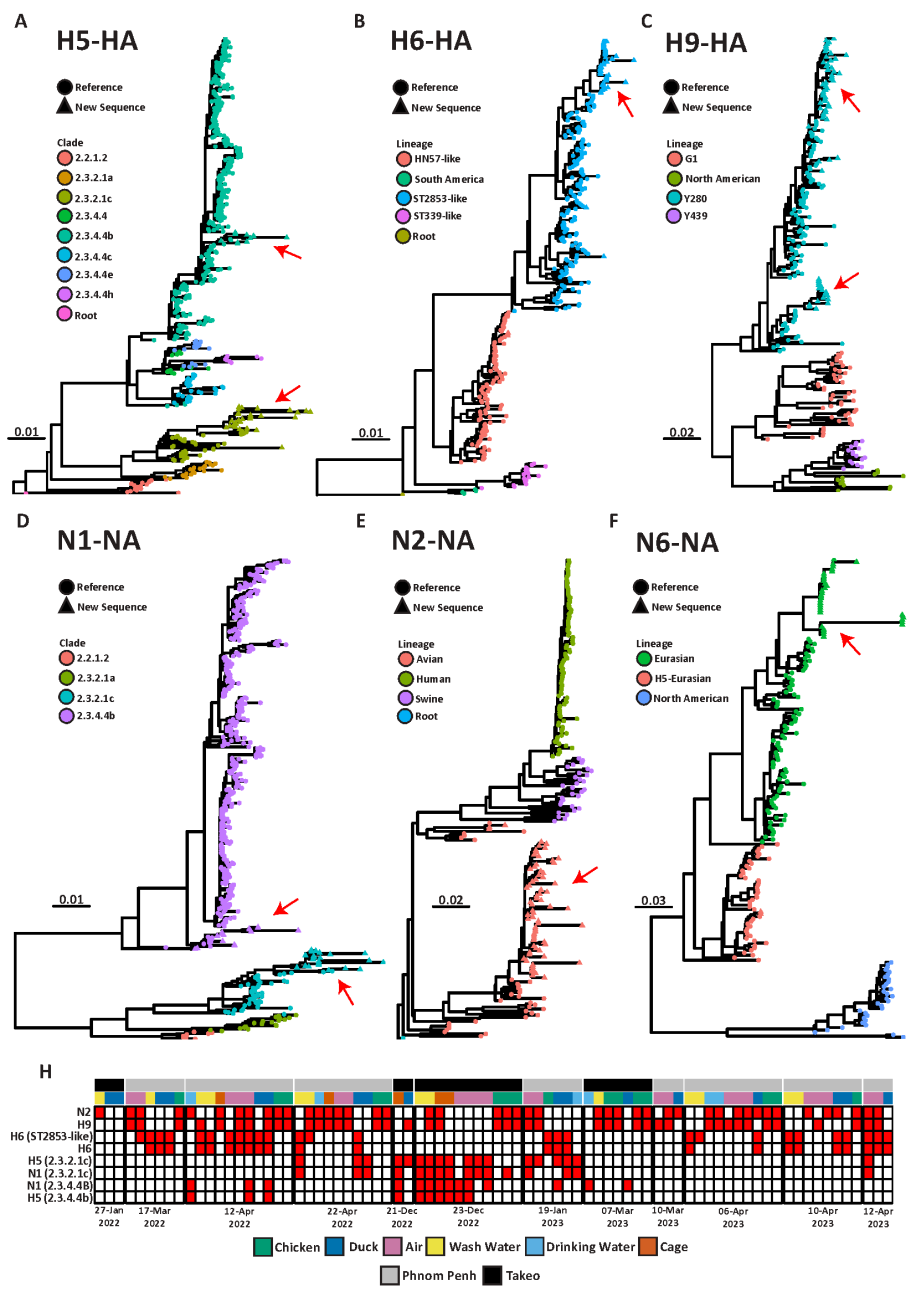
Environmental sampling detected highly pathogenic *Influenza A* contigs more often than poultry swabs. A-C) Maximum likelihood phylogenies of the HA subtypes A) H5, B) H6 and C) H9. D) Heatmap showing the detection rate of different HA and NA subtypes across all twelve visits to the respective markets. Ward.d2 hierarchal clustering reveals HA and NA subtype combinations which co-circulated. E-G) Maximum likelihood phylogenies NA *Influenza* subtypes D) N1, E) N2, F) N6. Triangle shaped tips show new sequences generated in the current study from one of two LBMs in Cambodia (which are also highlighted with a red arrow). Circle shaped tips indicate reference sequences. Each tip is coloured based on clade designation shown in the legend accompanying each phylogenetic tree.

Across the length of the sampling period, at both LBM locations, we identified four different influenza hemagglutinin (HA) subtypes in ducks and chickens including H5, H6, H7 and H9 (Fig. 3A–C and Supplementary Fig. 9–13). H5 clade 2.3.4.4b was found on three separate visits to the market (Fig. 3A, 3D and Supplementary Fig. 9). However, only once was this H5 clade identified through both traditional poultry swab sampling and corresponding ES, while 2.3.4.4b was found in wash water, cage swabs, and air samples at two other dates independent of avian sampling (Fig. 3D and Supplementary Fig. 9). Similar findings occurred for clade 2.3.2.1c, which was detected at LBMs on four separate sampling visits (Fig. 3A, 3D and Supplementary Fig. 10). On one occasion, both clade 2.3.2.1c and 2.3.4.4b were detected in eight different ES (4 air, 2 cage swabs and 2 wash water samples), suggesting high prevalence in birds coming into the market, and significant environmental contamination (Fig. 3D). However, no sequences were identified through direct animal sampling on this occasion, highlighting the versatility of environmental sampling.

In total, HA subtype H6 was found in 27 samples across the study, with both the ST2853-like and HN57-like clade detected. The HN57-like clade was found in ducks but was not detected in corresponding ES (Fig. 3B, 3D and Supplementary Fig. 12), and this was the only detection of the HN57-like clade of H6 influenza in this study. On six separate sampling visits, the ST2853-like H6 clade was successfully sequenced from poultry swabs as well as air samples and carcass wash water (Fig. 3B, 3D and Supplementary Fig. 11). On one visit, H6 was detected in air but not in the corresponding poultry swabs (Fig. 3D). The Eurasian avian lineage of H7 HA was only found once and was sequenced from both air and poultry samples at the same time and place (Supplementary Figs. 13 and 14). H9 HA was the predominant HA subtype detected, with a total of 43 positive samples across all 12 visits (Fig. 3C and Supplementary Figs. 15 and 16). On seven different sampling visits, H9 was identified in both poultry samples and ES. Importantly, on four separate sampling visits, H9 sequences were found in data from ES but were not identified in data from poultry swabs (Fig. 3D). The opposite trend was also reported, whereby H9 sequences were detected in duck samples but were not observed in any ES (N = 1). Overall, metagenomics using ES at LBMs detected circulating influenza A subtypes more often than metagenomics using traditional poultry swabs.

Three different neuraminidase (NA) gene subtypes were identified at LBMs in this study including N1, N2 and N6 (Fig. 3D–F). In line with H5 HA results, we identified both the 2.3.4.4b and the 2.3.2.1c clade of N1 (Fig. 3E and Supplementary Fig. 17–19). The 2.3.4.4b N1 clade was only detected in poultry swabs and ES (drinking water and air) on two visits (Fig. 3D and Supplementary Figs. 17 and 18). Six different ES yielded the detection of both H5 and N1 genes (representing the 2.3.4.4b and 2.3.2.1c clades) on December 23, 2022, at the LBM in Takeo province. N2 was the most common NA subtype found throughout the study and was consistently co-detected in both poultry swabs and ES (Fig. 3D, 3F and Supplementary Fig. 20). In line with other findings, N6 sequences of the H5 Eurasian lineage were also consistently co-detected in both poultry samples and ES (Fig. 3D, 3G and Supplementary Figs. 22 and 23). To further confirm the presence of full genomes of influenza A in our dataset, two internal genes were selected for analysis (PB2 and PA). In line with the findings for HA and NA, both PB2 and PA sequences *de novo* assembled from ES corresponded to those found in poultry swabs at each sampling timepoint (Supplementary Figs. 24 and 25).

Hierarchal clustering based on influenza HA and NA subtype detection revealed that the HA H9 commonly co-occurred with N2 at both LBMs (Fig. 3H). Using this approach, we were also able to infer that HA H6 (ST2853-like clade) co-occurred mostly with N6 while both H5 clades 2.3.2.1c and 2.3.4.4b co-occurred with N1 clades of the same subtype. While by nature metagenomic sequencing and *de novo* assembly of viral influenza A contigs from the environment and pooled poultry samples makes full length genome identification impossible for segmented RNA viruses, hierarchal clustering of detection patterns allows us to infer (co)-circulating subtypes.

### Environmental sample-derived contigs representing important avian pathogens are related to those identified in poultry swabs

**Figure 4:**
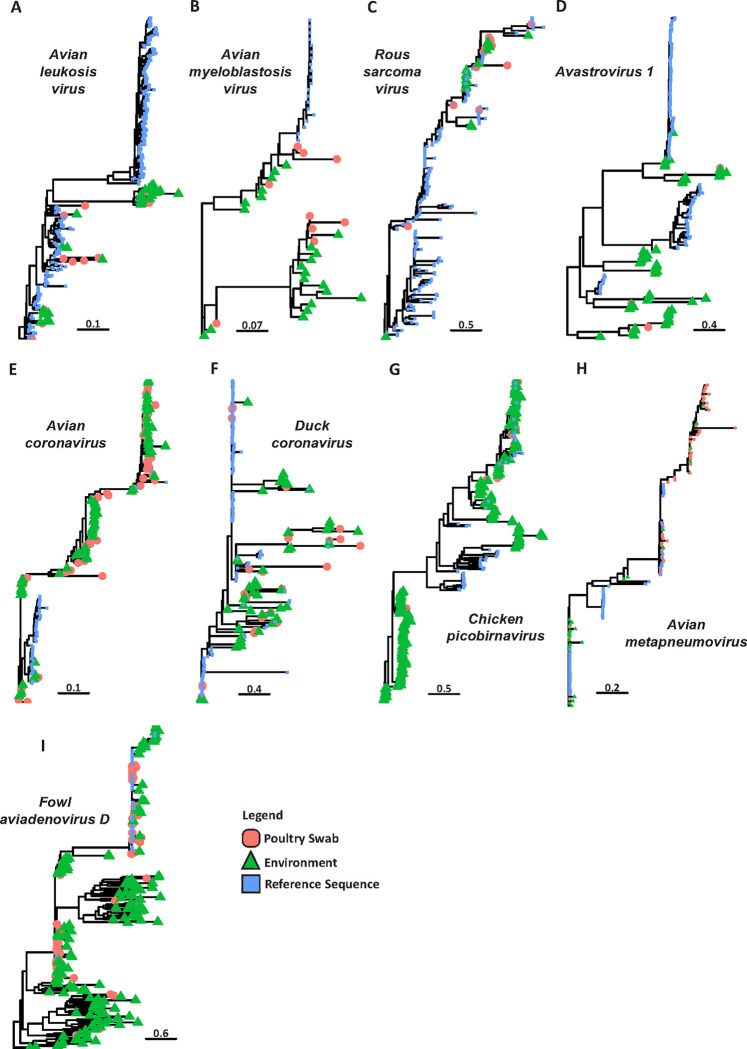
Environmental derived pathogen contigs are a short phylogenetic distance from those identified in poultry swabs. Phylogenetic tree reconstruction of nine avian pathogens successfully detected using environmental metagenomic surveillance inferred using the maximum likelihood method in IQ-TREE. Sequence type is determined by colour and shape of the node. Novel sequences assembled from the environment are shown in green (triangle) while sequences derived from poultry swabs are shown in red (circle). Reference sequences download from NCBI Virus Database are shown in blue (square). All trees represent full length genomes from the respective virus.

Given that *de novo* assembled influenza sequences from ES accurately represent those detected in poultry swabs, we assessed if environmentally-derived sequences from other viruses also represented those identified from poultry sampling. Phylogenetic trees were constructed for nine different pathogenic avian viruses within the families *Astroviridae*, *Coronaviridae*, *Adenoviridae*, *Retroviridae*, *Pneumoviridae* and *Picobirnaviridae*. The phylogeny of common oncogenic pathogens in the genus *Alpharetrovirus (family Retroviridae)* highlighted that sequences from poultry swabs were a short phylogenetic distance from ES sequences for three different viruses, namely, Avian leukosis virus, Avian myeloblastosis virus, and Rous sarcoma virus (Fig. 4A–C). This observation was also confirmed for Avastrovirus 1; however, ES also resulted in a greater number of contigs representing this virus than poultry swabs. Sequences from two different *Gamacoronaviruses (*Avian coronavirus and Duck coronavirus*)* also exhibited high similarity between poultry and environmental contigs (Fig. 4E–F). Importantly, the performance of ES was consistent regardless of which clade poultry swab derived contigs placed in the tree. Further analysis of both chicken picobirnavirus and a member of the *Pneumoviridae* family (Avian metapneumovirus) highlight ES as a robust method for accurately capturing avian viral sequences (Fig. 4G–H). For the virus Fowl aviadenovirus D, we report a large number of sequences obtained from the LBMs that group into a distinct clade, separate from the majority of available reference sequences (Fig. 4I). However, phylogenetic inference for Fowl aviadenovirus D again shows the that ES viral contigs are highly similar to those from traditional poultry swab sampling (Fig. 4I). Overall, these findings highlight the performance of environmental metagenomic surveillance as a tool for accurate detection of poultry pathogens.

## Discussion

Here we demonstrate that using targeted probe-enriched metagenomics on environmental surveillance samples can be an effective approach to replace targeted probe-enriched metagenomics on direct poultry swab samples for detecting viral sequences at LBMs. We found a strong association between viruses identified through poultry swabs and those detected in the surrounding environment. Phylogenetic analyses revealed high sequence similarity between viral contigs assembled from both sample types. Notably, metagenomics using ES detected AIV H5 clades 2.3.2.1c and 2.3.4.4b more frequently than metagenomics using traditional swabs. The sampling and analysis methodologies described have the potential to enable faster responses to disease outbreaks at LBMs, offering a more efficient and cost-effective alternative to conventional pathogen surveillance, especially in high-risk settings such as LBMs.

Previous work on pandemic respiratory viruses, including influenza A viruses and SARS-CoV-2, has shown that these viruses can remain infectious for extended periods outside the host (60–62), surviving in various environmental conditions and mediums such as air (63–67), inanimate surfaces (63, 68–70), and wastewater (71–74). While similar environmental persistence has been reported for AIVs, limited information is available about other avian pathogens (75, 76). In this study, detection of multiple avian pathogens from several viral families by metagenomics in the LBM environment demonstrates that ES is a useful method detection of sample collection in such settings. Furthermore, analysis of ES facilitated the detection of over 50 viruses that were not found in poultry swabs. While poultry may have been the host for some for these viruses, the majority of viruses may be from alternative sources/hosts yet still pose a significant threat to human and animal health. Importantly, this finding highlights the expanded surveillance coverage provided by ES, allowing the identification of pathogens which may otherwise go undetected.

Our findings show that avian pathogen viral nucleic acids persist outside the host in LBMs, possibly increasing the risk of infection of naive poultry through indirect transmission routes. Airborne transmission emerged as a particularly concerning pathway, as air from slaughter and holding areas contained more sequences from poultry viruses than other ES. Moreover, air samples collected near food stalls adjacent to the poultry processing area also contained avian viral sequences. These results imply that market employees, consumers, and bystanders may be at increased risk of infection via airborne transmission. These findings emphasize the argument for improved air quality and the physical separation of slaughter areas to mitigate the potential spread of poultry viruses. However, further studies are needed to determine if the nucleic acids detected in environmental samples represent viable, infectious viruses capable of replication.

Although each individual ES yielded fewer viruses compared to poultry swabs, ES exhibited high specificity in detecting viruses from different poultry swab types. For instance, chicken pathogens were strongly associated with air, cage swabs, and drinking water, while duck pathogens were more often detected in carcass wash water and oropharyngeal swabs. These results highlight the need for sampling diverse environmental types to maximize pathogen detection efficiency, particularly in markets with multiple bird species are present at different densities/quantities.

LBMs typically experience rapid turnover of poultry, with new batches arriving daily from multiple suppliers. Traditional random poultry sampling methods often fail to capture the full range of circulating viruses, as they rely on the assumption that sampled birds are infected. In contrast, our study shows that ES detects AIVs more frequently than traditional sampling. Despite the use of probe hybridization to enrich viral nucleic acids and improve sensitivity, some low-abundance taxa found in poultry swabs were undetected in environmental samples. These viruses were rare in the LBM during the study period, suggesting that low infection and replication rates may limit their environmental detection. Even with viral enrichment, some pathogens may fall below the detection threshold in environmental samples (77, 78).

Undetected avian pathogens were primarily associated with the throat and cloacal virome of ducks, while ES was more effective at capturing chicken pathogens. This may in part be explained by a larger quantity of chickens being sold at the particular LBMs sampled (79). Another explanation for this may be variations in virus shedding patterns between species (80–82). Previous meta-analysis has shown that chickens shed higher concentrations of certain influenza subtypes and other viruses through the cloaca compared to ducks (81). Our study supports these findings, with the cloaca of chickens emerging as the predominant source of viral pathogens in ES.

Overall, ES offers a safer, more cost effective and comprehensive alternative to traditional sampling methods, reducing both biosafety risks and animal welfare concerns associated with pathogen monitoring. Indeed, fewer samples are needed to understand an overview of the market virome and viruses which are not present in randomly sampled poultry can be detected. The framework used in this study could be adapted for other human-animal interfaces to strengthen pathogen surveillance systems and mitigate disease risks in similar settings.

## Figures and Tables

**Table 1:** Sample sizes for domestic poultry and environmental sample types

Samples collected				
	n	Sample type		

Poultry	59			
		Throat	Cloacal	
Chicken	30	15	15	
Duck	29	14	15	

Environmental	82			
		Holding area	Slaughter area	Outide
Air	37	13	14	10

		Wash water	Drinking water	
Water	28	19	9	

Cage	17			

## Data Availability

Data used in this study is publicly available under accession number PRJEB83776.
